# Statistical models for the deterioration of kidney function in a primary care population: A retrospective database analysis

**DOI:** 10.12688/f1000research.20229.1

**Published:** 2019-09-10

**Authors:** Jason L Oke, Benjamin G Feakins, Iryna Schlackow, Borislava Mihaylova, Claire Simons, Chris A O'Callaghan, Daniel S Lasserson, F D Richard Hobbs, Richard J Stevens, Rafael Perera

**Affiliations:** 1 Nuffield Department of Primary Care Health Sciences, University of Oxford, Oxford, OX2 6GG, UK; 2 Nuffield Department of Population Health, University of Oxford, Oxford, OX3 7LF, UK; 3 Centre for Primary Care and Public Health, Queen Mary, University of London, London, E1 2AB, UK; 4 Nuffield Department of Medicine, University of Oxford, Oxford, OX3 7LF, UK; 5 Institute of Applied Health Research, University of Birmingham, Birmingham, B15 2TT, UK

**Keywords:** Kidney Function Decline, Chronic Kidney Disease (CKD), Estimated Glomerular Filtration Rate (eGFR), Proteinuria, Hidden Markov Model (HMM), Primary Care, Clinical Practice Research Datalink (CPRD)

## Abstract

**Background:** Evidence for kidney function monitoring intervals in primary care is weak, and based mainly on expert opinion. In the absence of trials of monitoring strategies, an approach combining a model for the natural history of kidney function over time combined with a cost-effectiveness analysis offers the most feasible approach for comparing the effects of monitoring under a variety of policies. This study aimed to create a model for kidney disease progression using routinely collected measures of kidney function.

**Methods:** This is an open cohort study of patients aged ≥18 years, registered at 643 UK general practices contributing to the Clinical Practice Research Datalink between 1 April 2005 and 31 March 2014. At study entry, no patients were kidney transplant donors or recipients, pregnant or on dialysis. Hidden Markov models for estimated glomerular filtration rate (eGFR) stage progression were fitted to four patient cohorts defined by baseline albuminuria stage; adjusted for sex, history of heart failure, cancer, hypertension and diabetes, annually updated for age.

**Results:** Of 1,973,068 patients, 1,921,949 had no recorded urine albumin at baseline, 37,947 had normoalbuminuria (<3mg/mmol), 10,248 had microalbuminuria (3–30mg/mmol), and 2,924 had macroalbuminuria (>30mg/mmol). Estimated annual transition probabilities were 0.75–1.3%, 1.5–2.5%, 3.4–5.4% and 3.1–11.9% for each cohort, respectively. Misclassification of eGFR stage was estimated to occur in 12.1% (95%CI: 11.9–12.2%) to 14.7% (95%CI: 14.1–15.3%) of tests. Male gender, cancer, heart failure and age were independently associated with declining renal function, whereas the impact of raised blood pressure and glucose on renal function was entirely predicted by albuminuria.

**Conclusions:** True kidney function deteriorates slowly over time, declining more sharply with elevated urine albumin, increasing age, heart failure, cancer and male gender. Consecutive eGFR measurements should be interpreted with caution as observed improvement or deterioration may be due to misclassification.

## Introduction

The National Institute for Health and Care Excellence recommend monitoring kidney function using estimated glomerular filtration rate (eGFR) in people with, or at risk of, chronic kidney disease (CKD) ^1^. The guideline suggests increasing the intensity of monitoring according to the current level of eGFR and albumin-creatinine ratio, stating that monitoring should be tailored according to i) the underlying cause of CKD and ii) past patterns of eGFR and albumin-creatinine ratio, comorbidities, changes to treatments such as reninangiotensin-aldosterone system antagonists, inter-current illness and whether the patient has chosen conservative management of CKD. One of the objectives of monitoring eGFR is to detect progression of CKD, which could precede end-stage renal disease (ESRD). ESRD is associated with substantial morbidity and mortality, with cardiovascular disease mortality rates 10 to 30 times higher in patients on dialysis than in the general population ^2^. Yet, kidney function declines slowly with age and ESRD is rare, even for people with moderately impaired renal function (eGFR 30–59 ml/min/1.73m ^2^). In a study of 58,000 people with CKD stage 3 who were followed for 10 years, the cumulative incidence was 40 per 1,000 people ^3^. It follows that recommendations to monitor everyone annually or more frequently in a community setting for progressive kidney function loss will have a poor yield. Furthermore, as eGFR is a noisy measurement, with a within-person coefficient of variation estimated to be approximately 5.5% ^4^, it is likely two consecutive eGFR measurements may appear to indicate declining renal function when underlying renal function is stable (false positive), or stable renal function when underlying renal function has deteriorated (false negative). Finally, it is arguable as to whether there are any actions that can be taken to halt the deterioration of renal function if progressive CKD is found, as there is currently very little evidence that “catching” CKD early produces any benefit ^5^.

There have been no trials of screening or monitoring for CKD ^6^ and recommendations for how frequently monitoring should take place are based on expert opinion. In the absence of trials, an approach combining a model for the natural history of kidney function over time combined with a cost-effectiveness analysis offers the most feasible approach for comparing the effects of monitoring under a variety of policies. The aim of this study was to create a model for kidney disease progression using routine measures of kidney function. Our approach simultaneously estimates the true rate of kidney function loss and the probability of misclassification that inevitably occurs from using eGFR. Our study is conducted in a general primary care population and our results will be useful in guiding future recommendations for the timing of monitoring eGFR in primary care.

## Methods

### Ethical statement

The protocol for this research was approved by the Independent Scientific Advisory Committee of the Medicines and Healthcare Products Regulatory Agency (protocol number 14_150R). Ethical approval for observational research using the Clinical Practice Research Datalink with approval from the Independent Scientific Adisory Committee has been granted by a National Research Ethics Service committee (Trent Multi Research Ethics Committee, REC reference number 05/MRE04/87).

### Source and selection of participants

We used the UK Clinical Practice Research Practice Datalink (CPRD) ^7^ to construct an open cohort of adults (≥18 years of age) registered at practices deemed to have “acceptable” patient records (termed “up-to-standard” in CPRD). We included patient records starting from 1 April 2005, post-dating the publication of the Kidney Disease Outcomes Quality Initiative (KDOQI) guidelines for the classification of CKD in 2002 ^8^ and the introduction of Quality and Outcomes Framework targets in UK primary care in 2004. The study end date was 31 March 2014. Eligible patients had to be registered with their practice for a minimum of 12 months before study entry to ensure adequate recording of baseline covariates. We excluded patients who, in the 12 months before study entry, were pregnant, were receiving dialysis, or were living kidney donors or recipients. Follow-up ended at the study end date, unless preceded by the date of death, transfer out of CPRD, the last available linked data, or (where applicable) pregnancy, renal transplantation/donation, or dialysis.

### Statistical analysis

To model decline in kidney function, hidden Markov models (HMMs) ^9– 13^ were fitted to four patient cohorts defined by baseline albuminuria stage: 1) no albuminuria measurement (unmeasured), 2) normoalbuminuria (<3 mg/mmol), 3) microalbuminuria (3–30 mg/mmol), and 4) macroalbuminuria (>30 mg/mmol). Models were adjusted for sex, heart failure, cancer, hypertension and diabetes, and annually updated age.

**Figure 1.**
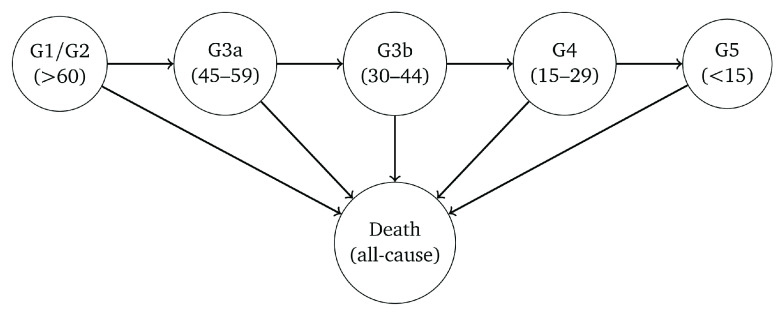
Representation of the model for the deterioration of kidney function over time. Arrows indicate permitted (instantaneous) transitions. The numbers in brackets depict the estimated glomerular filtration rate ranges (in ml/min/1.73 ^2^) associated with each stage.

The HMMs comprised two components, a multi-state model governing the ‘true’ underlying progression of CKD, and a second model for the probability of misclassification to allow for the variability in eGFR. The underlying model for CKD was parametrised as uni-directional, in which true kidney function could only deteriorate over time (no spontaneous improvement). The outcome was eGFR stage based on the criteria used for the diagnosis of CKD, i.e. G1–G5. We combined stages G1 and G2 for the purposes of improving model fit. Death from any cause was assumed to be an absorbing state. A representation of the HMMs is depicted in Figure 1.

The HMMs were specified so that it was possible for misclassification to occur in neighbouring eGFR categories. Hence, for a person with true GFR >60 ml/min/1.73m ^2^ we specified the model so that a single measurement of eGFR could fall within a G3a or G3b category due to measurement error and biological variation, but not G4 or G5. For a person with true eGFR in stage G3b, a single measurement of eGFR could be misclassified as either G1/2, G3a, G4 or G5. Death was the only state assumed to be always classified correctly.

To assess model fit, we used a split-sample approach. Although this is a weak procedure for low-variance methods, such as the Cox proportional hazards model or logistic regression, it is useful for a model that can be over-parametrised or exhibit convergence issues (such as a HMM). We split the data using pseudo-random numbers into equal size training and testing data sets. The model was fit in the training data set and then used to predict trajectories of eGFR for patients in the testing data set, based on their measurement times and covariates. Calibration plots were used to compare the predicted and observed proportion of tests falling within each eGFR category over time. Annual transition rates for kidney function loss and death from any cause were estimated from the model, along with the misclassification probabilities and transition rate multipliers for age, sex, heart failure and cancer, and presented as state model diagrams. The models were used to estimate the probability of progression to a higher stage within six, 12 or 36 months, along with the probability that an eGFR test taken at that time would detect the change (true positive), and the probability that a change in eGFR stage would occur in a person in whom true kidney function had not changed (false positive), for all cohorts for baseline stages G3a and G3b; see Supplementary Tables S18–21 ( *Extended data*) ^14^.

Finally, we estimated global misclassification probabilities for the four cohorts using the Viterbi algorithm ^15^ to find the underlying sequence of true eGFR stages with the highest probability given the observed sequence. Assuming the state predicted by the model was the truth, we calculated the proportion of times the observed state was a lower stage than predicted (under-grading) and the proportion of times the observed was a higher stage than predicted (over-grading), and then added these together to calculate the total number of misclassified tests across cohorts.

All analyses were performed in R version 3.6.1 (“Action of the Toes”) ^16^, with HMMs fit using version 1.6.7 of the *msm* package ^17^. Scripts used in these analyses are available (see *Software availability* ) ^18^.

## Results

The initial data set comprised 3,338,526 patients. A total of 1,365,458 patients whose records contained fewer than three eGFR tests were excluded, leaving 1,973,068 patients eligible for analysis: 1,921,949 without a urine albumin test on record, 37,947 with normoalbuminuria (<3 mg/mmol), 10,248 with microalbuminuria (3–30 mg/mmol), and 2,924 with macroalbuminuria (>30 mg/mmol). Each of the four cohorts were split into two halves and nominated as training and testing data sets. Due to the computational demands of the statistical method used, we randomly selected a sub-cohort of 50,000 patients to fit the model in the cohort without a urine albumin test on record. Summary statistics of patient characteristics from the four cohorts are presented in Table 1.

**Table 1.** Patient characteristics at baseline, by albuminuria stage.

Variable	Category	Albuminuria Stage, Number (%)			
		Unmeasured	Normoalbuminuria	Microalbuminuria	Macroalbuminuria
	Total	1,921,949 (100.0%)	37,947 (100.0%)	10,248 (100.0%)	2,924 (100.0%)
Gender	Female Male	1,058,400 (55.1%) 863,549 (44.9%)	18,312 (48.3%) 19,635 (51.7%)	4,749 (46.3%) 5,499 (53.7%)	1,352 (46.2%) 1,572 (53.8%)
Age (years)	18–39 40–49 50–59 60–69 70–79 80–89 90+	254,037 (13.2%) 324,362 (16.9%) 419,561 (21.8%) 421,704 (21.9%) 321,522 (16.7%) 154,881 (8.1%) 25,882 (1.3%)	2,701 (7.1%) 4,811 (12.7%) 7,354 (19.4%) 9,930 (26.2%) 8,610 (22.7%) 3,948 (10.4%) 593 (1.6%)	502 (4.9%) 928 (9.1%) 1,547 (15.1%) 2,191 (21.4%) 2,575 (25.1%) 2,006 (19.6%) 499 (4.9%)	267 (9.1%) 352 (12.0%) 514 (17.6%) 638 (21.8%) 602 (20.6%) 444 (15.2%) 107 (3.7%)
Ethnicity	Missing White Black Asian Mixed Other	1,133,893 (59.0%) 461,796 (24.0%) 28,480 (1.5%) 12,314 (0.6%) 276,750 (14.4%) 8,716 (0.5%)	18,469 (48.7%) 10,077 (26.6%) 1,325 (3.5%) 728 (1.9%) 6,999 (18.4%) 349 (0.9%)	4,950 (48.3%) 2,465 (24.1%) 512 (5.0%) 229 (2.2%) 1,958 (19.1%) 134 (1.3%)	1,821 (62.3%) 515 (17.6%) 106 (3.6%) 47 (1.6%) 422 (14.4%) 13 (0.4%)
eGFR (ml/min/1.73m ^2^)	>60 45–59 30–44 15–29 <15	1,524,003 (79.3%) 295,312 (15.4%) 85,303 (4.4%) 16,091 (0.8%) 1,240 (0.1%)	27,753 (73.1%) 6,850 (18.1%) 2,724 (7.2%) 591 (1.6%) 29 (0.1%)	6,114 (59.7%) 2,147 (21.0%) 1,440 (14.1%) 518 (5.1%) 29 (0.3%)	1,628 (55.7%) 569 (19.5%) 453 (15.5%) 247 (8.4%) 27 (0.9%)
CKD Read Code	None G1/2 G3 G4 G5	1,911,565 (99.5%) 2,660 (0.1%) 7,347 (0.4%) 457 (0.0%) 87 (0.0%)	37,521 (98.9%) 122 (0.3%) 282 (0.7%) 21 (0.1%) 1 (0.0%)	10,044 (98.0%) 23 (0.2%) 148 (1.4%) 34 (0.3%) 0 (0.0%)	2,870 (98.2%) 5 (0.2%) 31 (1.1%) 17 (0.6%) 2 (0.1%)
Cancer	No Yes	1,884,014 (98.0%) 37,935 (2.0%)	37,698 (99.3%) 249 (0.7%)	10,206 (99.6%) 42 (0.4%)	2,912 (99.6%) 12 (0.4%)
Chronic Renal Disease	No Yes	1,919,946 (99.9%) 2,003 (0.1%)	37,928 (99.9%) 19 (0.1%)	10,230 (99.8%) 18 (0.2%)	2,914 (99.7%) 10 (0.3%)
Diabetes	No Yes	1,866,051 (97.1%) 55,898 (2.9%)	35,850 (94.5%) 2,097 (5.5%)	9,660 (94.3%) 588 (5.7%)	2,810 (96.1%) 114 (3.9%)
Heart Failure	No Yes	1,905,724 (99.2%) 16,225 (0.8%)	37,778 (99.6%) 169 (0.4%)	10,209 (99.6%) 39 (0.4%)	2,914 (99.7%) 10 (0.3%)
Hypertension	No Yes	1,512,801 (78.7%) 409,148 (21.3%)	34,353 (90.5%) 3,594 (9.5%)	9,541 (93.1%) 707 (6.9%)	2,749 (94.0%) 175 (6.0%)
Ischaemic Heart Disease	No Yes	1,841,610 (95.8%) 80,339 (4.2%)	37,275 (98.2%) 672 (1.8%)	10,122 (98.8%) 126 (1.2%)	2,897 (99.1%) 27 (0.9%)
Peripheral Vascular Disease	No Yes	1,895,750 (98.6%) 26,199 (1.4%)	37,766 (99.5%) 181 (0.5%)	10,213 (99.7%) 35 (0.3%)	2,912 (99.6%) 12 (0.4%)
Stroke or TIA	No Yes	1,890,775 (98.4%) 31,174 (1.6%)	37,695 (99.3%) 252 (0.7%)	10,189 (99.4%) 59 (0.6%)	2,905 (99.4%) 19 (0.6%)

Six state continuous time HMMs adjusted for sex, heart failure, cancer, hypertension and diabetes, and annually updated age were fit on the four training data sets. Hypertension and diabetes were subsequently removed from the models as they were unable to predict eGFR stage progression or death. All models converged to their respective maximum likelihood estimates, with positive definitive Hessian matrices permitting confidence interval estimation for all parameters. Intensity, transition and misclassification matrices for these models are given in Supplementary Tables S2–13 ( *Extended data*) ^14^.

**Figure 2.**
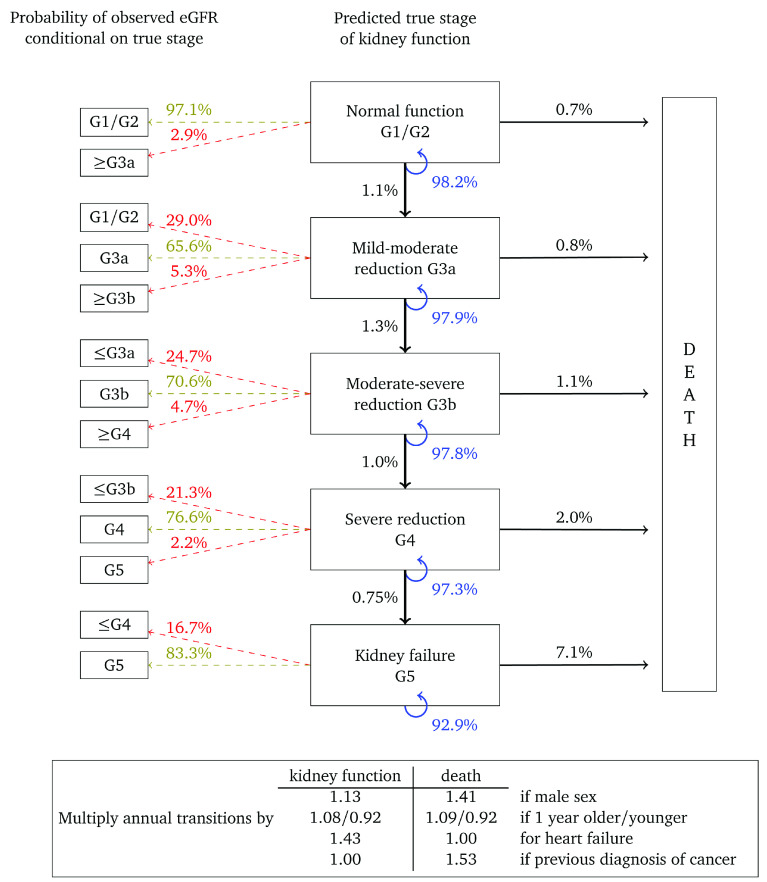
Annual transition model diagram for patients with unmeasured urine albumin at baseline. Probabilities are based on a woman aged 60, without heart failure or a previous diagnosis of cancer.

Figure 2 shows the annual transition and misclassification probabilities for a woman, aged 60, without heart failure or a previous diagnosis of cancer and with no urine albumin test on record. The figure shows that if kidney function is normal (G1/G2) then the probability of her true kidney function deteriorating to stage G3a in one year is estimated to be 1.1%. The probability that a single eGFR test will be misclassified as G3a is 2.9%, while the probability that it will correspond to her true stage is 97.1%. The probability that this woman dies within a year is estimated to be 0.7%. The probability that her kidney function remains in this category is 98.2%. If the woman is one year older then transition probabilities should be multiplied by 1.08 for kidney function and 1.09 for death. For example, the annual transition probability from stage G3b, is 1.0% for a 60 year old woman, but 1.0 *×* 1.08 ^10^ = 2.16% for a 70 year old woman and *×* 1.08 ^20^ = 4.66% for woman who is 80 years old. Multipliers in which the confidence interval overlapped “no effect” are set to 1.00.

**Figure 3.**
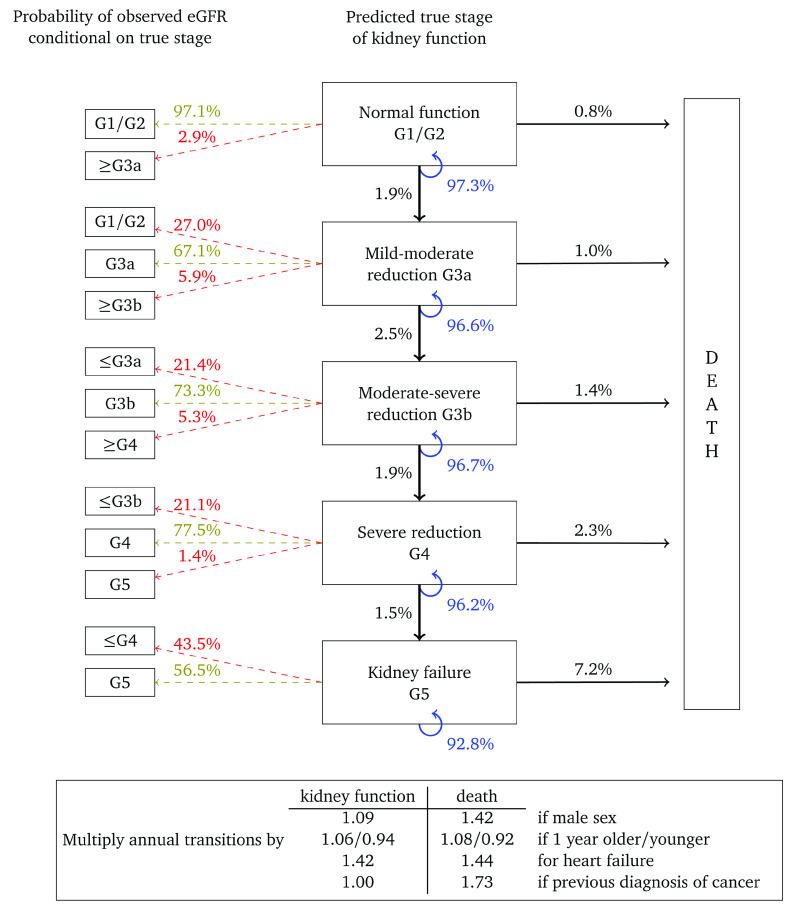
Annual transition model diagram for patients with normoalbuminuria at baseline. Probabilities are based on a woman aged 60, without heart failure or a previous diagnosis of cancer.

Figure 3 represents annual transitions for a woman with the same characteristics, but who has had her urine albumin tested and found to be in the normoalbuminuric range. Corresponding annual transition probabilities for kidney function are nearly twice that of an equivalent woman without a urine albumin test on record. Respective transition rates to death from each stage are also higher, illustrating that this cohort represents women in poorer health. Misclassification probabilities and transition probability multipliers are broadly similar to Figure 2.

**Figure 4.**
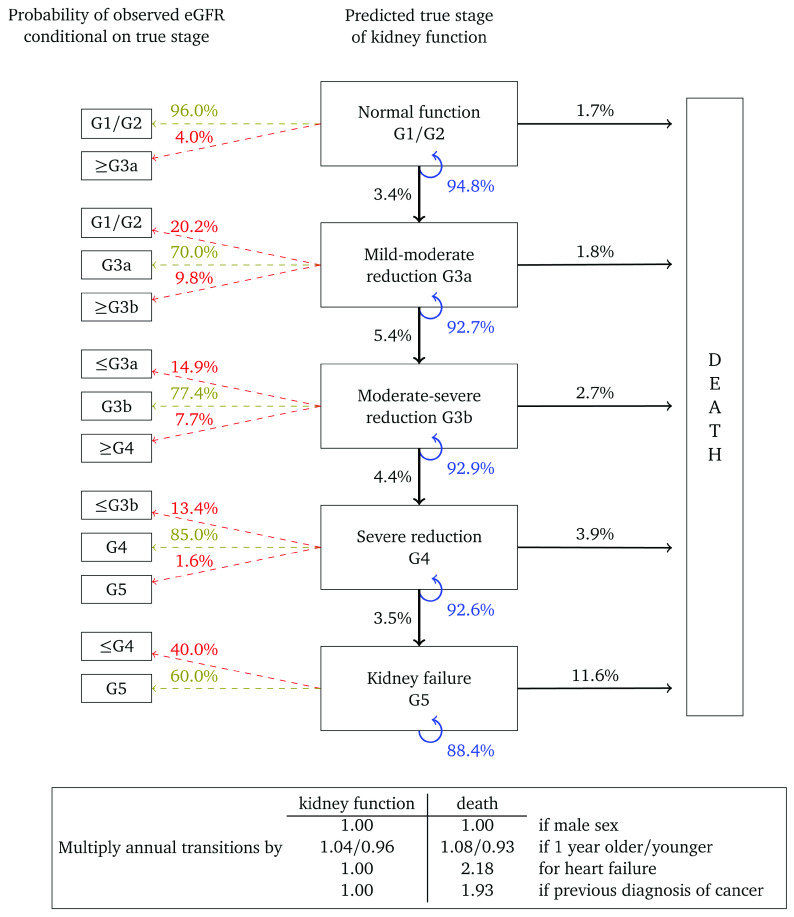
Annual transition model diagram for patients with microalbuminuria at baseline. Probabilities are based on a woman aged 60, without heart failure or a previous diagnosis of cancer.

**Figure 5.**
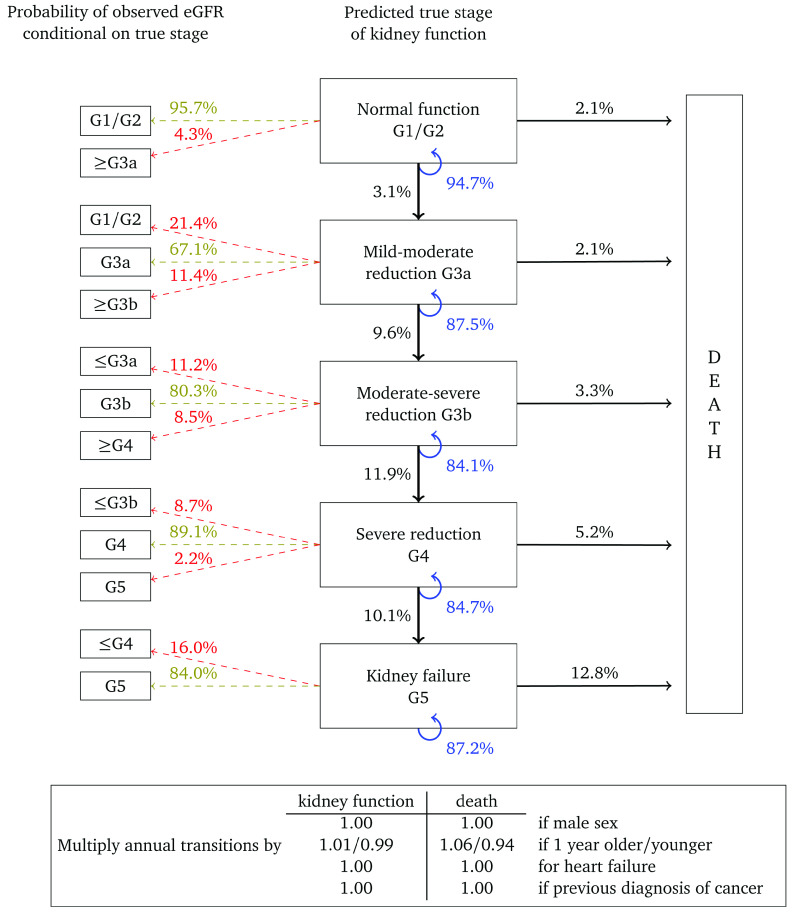
Annual transition model diagram for patients with macroalbuminuria at baseline. Probabilities are based on a woman aged 60, without heart failure or a previous diagnosis of cancer.

Figure 4 and Figure 5 show results for women with micro- and macroalbuminuria, respectively. Kidney function transition probabilities are higher, as are annual transition probabilities for death. Fewer transition multipliers are significant for these cohorts but this probably reflects the smaller cohort sizes and correspondingly reduced statistical power.

Table 2 shows the results from applying the Viterbi algorithm to the four cohorts. Under-grading of eGFR stage occurs more often than over-grading in all cohorts but over-grading tends to increase for cohorts having urine albumin tests. In total, 12.1% (11.9–12.2%) of all tests done in the unmeasured urine albumin cohort are misclassified, 13.1% (13.0–13.3%) in patients with normoalbuminuria, 14.5% (14.2–14.8%) in patients with microalbuminuria, and 14.7% (14.1–15.3%) in patients with macroalbuminuria.

**Table 2.** Probability (%) that any eGFR test is under-graded and/or over-graded, by albuminuria stage. 95% confidence intervals shown in brackets.

Grading	Unmeasured	Normoalbuminuria	Microalbuminuria	Macroalbuminuria
Under-grading	8.3 (8.2–8.5)	9.2 (9.0–9.3)	8.7 (8.5–8.9)	8.5 (8.1–9.0)
Over-grading	3.7 (3.6–3.8)	4.0 (3.9–4.0)	5.8 (5.6–6.0)	6.2 (5.8–6.6)
Total	12.1 (11.9–12.2)	13.1 (13.0–13.3)	14.5 (14.2–14.8)	14.7 (14.1–15.3)

Mean sojourn time, i.e. the average time spent in each state, decreased with increasing severity of eGFR and albuminuria stage ( Table 3). One exception was for macroalbuminuric patients in eGFR stage G5, for whom the mean sojourn time was greater than for microalbuminuric patients in eGFR stage G5. However, few patients were present in the more severe diseases states and the 95% confidence intervals of the two estimates substantially overlap.

**Table 3.** Mean sojourn times, by albuminuria and eGFR stage. 95% confidence intervals shown in brackets.

eGFR Stage	Unmeasured	Normoalbuminuria	Microalbuminuria	Macroalbuminuria
G1/2	30.5 (29.2–31.8)	20.1 (19.3–20.9)	10.4 (9.7–11.1)	13.1 (11.3–15.3)
G3a	26.7 (25.2–28.4)	15.7 (14.9–16.6)	7.5 (6.9–8.1)	6.0 (5.1–7.0)
G3b	25.1 (23.3–27.1)	15.7 (14.6–16.8)	7.3 (6.7–8.1)	4.5 (3.9–5.3)
G4	19.5 (17.4–21.8)	12.8 (11.5–14.3)	6.8 (5.9–7.7)	4.5 (3.7–5.5)
G5	7.0 (5.6–8.9)	5.9 (4.6–7.6)	3.6 (2.8–4.7)	4.2 (3.1–5.7)

## Discussion

We have developed a statistical model for kidney function monitoring over time, using a large clinical database of longitudinal kidney function measurements from an unselected primary care cohort. This model takes into account that observed kidney function is measured with error and uses statistical methodology to estimate the underlying ‘true’ rate of progression. We stratified our models by albuminuria stage in accordance with the findings of previous studies that showed that urine albumin excretion is a significant risk factor for the progression of CKD and the development of ESRD ^19– 21^. Our analyses suggest that kidney function declines more rapidly in men than in women, independent of other risk factors. Existing evidence for differences in the rates of progression between men and women is conflicting ^3, 22, 23^. Our analysis supports the observations of others, that men are over-represented in the latter stages of CKD ^24^, with our model predicting a slower progression of kidney disease for women in the unmeasured urine albumin and normoalbuminuria cohorts. The fact that women are over-represented at CKD stage 3 may be due to the fact that women tend to live longer than men.

We estimated the probability of misclassification conditioning on true eGFR stage. A consistent pattern is seen across the different baseline urine albumin levels and by eGFR stage. Our model suggests that on average, change in underlying kidney function is slow with mean sojourn times in stage G3a and G3b being between 15 and 25 years for patients without elevated urine albumin. Given the slow rate of change and the high chance that observed eGFR misclassifies the true eGFR stage, frequent testing of eGFR in these populations will inevitably lead to the detection of more spurious change than real change.

We assessed whether our models of kidney disease progression would be improved by adjusting for clinical characteristics that were *a priori* considered to be associated with increased risk, and therefore, faster progression. Our analysis did not support the notion that diabetes, hypertension, peripheral vascular disease, ischaemic heart disease, stroke or transient ischaemic attack are independently associated with deterioration of kidney function once albuminuria stage and updated eGFR are accounted for. We conclude that conditioning on eGFR stage and urine albumin levels, knowledge of diabetes status is less important, but we cannot rule out that our study may be under-powered to detect small but real effects on transition rates.

A major strength of this study is that we have taken a very large and unselected sample of patients from a database that has been shown to be representative of the wider UK population ^7^. Our model for progression takes into account multiple stages of kidney function and the competing risk of death from any cause. We have also employed a method that takes into account that eGFR is observed with error, and simultaneously estimates true underlying eGFR ^25^. This means that we can estimate misclassification probabilities and evaluate the effects of different monitoring strategies. We used a split-sample approach to assess for potential over-fitting and the internal validity of the model.

Our study has a number of limitations. Our data was not collected for the purpose of conducting a study about modelling progression of kidney function. As a consequence, we do not know the reasons tests were conducted, and for many patients, records were incomplete and examination times were irregular. The extent to which this could bias our findings is unclear as it depends on our understanding of the examination scheme used by the doctors. We recognise three potential mechanisms for these tests to occur in a primary care setting. A significant number of creatinine tests will be ‘random’ with respect to the kidney function, because they would have been ordered as part of a routine check-up and not specifically to monitor or diagnose kidney disease. This could be a result of the co-reporting of serum creatinine as part of ‘test batches’ in which other biomarkers would have been of primary interest, or because serum creatinine may have been requested prior to the initiation of a potentially nephrotoxic drug. For some patients, the timing of the next measurement will have been influenced by the current kidney function level. This is likely to have happened if the purpose of the test is to monitor CKD and current clinical guidelines are followed ^1^. This mechanism has been referred to as ‘doctor’s care’ in the literature. The third scenario is when a patient initiates the timing of their test themselves, so called ‘patient self-selection’. Of the three scenarios, we consider the self-selection scenario possible but less likely than the other schemes due to the asymptomatic nature of kidney function loss in all but the end-stages of the disease. Grüger *et al*. ^26^ showed that estimated transition rates are only biased under the “patient self-selection” examination schemes and transition rate estimates are unbiased if inefficient under doctor’s care scheme. In the case of random timing, the estimates are both efficient and unbiased.

We attempted to include a state to represent transient and acute loss of kidney function (acute kidney injury) as this is a contributing factor to CKD, but the addition of this non-absorbing state with pathways back to each state resulted in over-parametrisation of the model. Furthermore, data on urine albumin, body mass index and ethnicity is missing in a large number of patients in CPRD. To overcome this, we created a sub-group of patients in whom urine albumin was not recorded. The omission of ethnicity in this model is a limitation as kidney function decline is considered to differ between ethnic groups. We were not able to adjust our models for ethnicity, as historically, ethnicity has been poorly recorded in CPRD.

It is likely that once a patient’s kidney function has been observed in stage 4 or 5, they are referred to specialist care, with subsequent kidney function testing occurring outside the CPRD database. Hence, these patients’ records are missing from our study, which potentially explains why transition rates slow down rather than increase, as might be expected. In a study of electronic health records data from Pennsylvania, a similar model was fit to eGFR records, and reported that transition probabilities between kidney function stages generally increased as stage increased for all but stage 3 ^25^. Even so, our model calibrates well with reports of progression to ESRD from different stages. For example, Tangri *et al*. ^27^ reported that three from 2,014 people with CKD stage 3 at baseline progressed to ESRD after three years of follow-up. Assuming this population contained an equal proportion of people with CKD stage 3a and 3b, then our model, based on the unmeasured urine albumin cohort, would predict that just one person would reach stage 5 after three years. Using the model for patients with normoalbuminuria, it would be three people. From the same study, 22 of 826 people progressed from stage 4 at baseline to kidney failure after three years. Our models predict 25 people with unmeasured urine albumin and 46 people with normoalbuminuria would reach stage 4. In a study reporting on sex differences in CKD progression, the rate of ESRD per 100 person-years was 3.1 in women and 3.8 in men. Based on our model for patients with normoalbuminuria our equivalent estimates are 1.9 and 2.3, but 2.07 and 2.13 for patients with microalbuminuria and 3.0 and 3.2 for patients with macroalbuminuria. Our study shows that kidney function deteriorates slowly in most patients with average sojourn times in decades rather than years. Whilst eGFR is widely used to measure kidney function we estimate that the potential for misclassification is large and clinically relevant, with implications for monitoring for rapid kidney function loss or pharmacovigilance. For example, of 1,741 people with CKD stage 3 recruited for a study from 32 primary care practices in the UK ^28^, 496 were in remission at baseline (although qualifying at the recruitment stage) and of these, 157 were back to CKD stage 3 at one year, with a further 132 returning to stage 3 CKD by five years. This type of pattern is consistent with our model, in which underlying kidney function only deteriorates but is observed with error. If our model is correct, then it is clear to see how monitoring CKD periodically will confuse and might lead to inappropriate action.

## Conclusions

We have developed a model to predict decline in kidney function and used it to assess different monitoring strategies and screening programmes. The model takes into account stage progression and test error, which were recently identified as important for future economic evaluations of CKD testing ^29^. Future work in this field could look to validate this model in another primary care population, ideally one in which patients are followed throughout including stages 4 and 5.

## Data availability

### Underlying data

The data used in this study are not publicly available and were obtained under licence. The terms of this license do not permit us to share the data. However, those wishing to replicate our analysis in this database can apply directly to the Medicines and Healthcare Products Regulatory Agency (MHRA) for access to the CPRD, at enquiries@cprd.com. The conditions under which the MHRA will grant access are beyond our control, but are explained at https://www.cprd.com/research-applications.

### Extended data

Figshare: Statistical models for the deterioration of kidney function in a primary care population: A retrospective database analysis (Extended Data). https://doi.org/10.6084/m9.figshare.9741611.v1 ^14^.

This project contains the following extended data:

- Extended Data.pdf (document containing Tables S1–20 and Figures S1–5)

Data are available under the terms of the Creative Commons Attribution 4.0 International license (CC-BY 4.0).

## Software availability

Source code available from: https://github.com/OxPrimaryCareStats/egfr-decline/tree/v1.0.0

Archived source code at time of publication: https://doi.org/10.5281/zenodo.3377113 ^18^

License: MIT License
