# Supplementary material for: Statistical models for the deterioration of kidney function in a primary care population: A retrospective database analysis
Source: F1000Res. 2022 Aug 8;8:1618. Originally published 2019 Sep 10. [Version 2] doi: 10.12688/f1000research.20229.2 (PMC9532959; doi:10.12688/f1000research.20229.2)
Supplement: Peer review history

# Peer review: Reviewer response for version 2

**DOI:** 10.5256/f1000research.136788.r146903

**Published:** 2022-10-04

**Authors:** Lawrence Hunsicker

**Affiliations:** 1 Department of Internal Medicine, University of Iowa, Iowa City, USA

The revised version includes no new or revised analyses.  So this Version 2 report deals entirely with the authors' responses to my Version 1 Report and the edits in their Discussion. I repeat the positive aspects of this manuscript summarised in the first two paragraphs of my Version 1 Report, dealing with the importance of the issue raised by the authors, the almost total absence of evidence on the utility of measurement of eGFR and proteinuria in a population of subjects similar to those followed in UK General Practices, the availability of the nearly unique set of data from the Clinical Practice Research Datalink, and the authors' competence in performing their Hidden Markov Model analysis.  My reservations dealt mostly with the authors' choice of analytic model.

 The authors' rationale for their use of their Hidden Markov Model is that it permits adjustment for the well-documented degree of CKD stage misclassification that is revealed when estimates of eGFR are compared with directly measured GFR (itself measured with significant "error" when evaluated by replicate measurements). But their Hidden Markov Model also makes assumptions that may well not hold in reality.  There are two specific issues with the authors' Markov assumptions: a. that eGFR once "lost" cannot be recovered, and b: that the history of prior changes in renal function is not informative once a Markov transition has occurred.  This model seems to be based on an assumption that reduction of eGFR is mostly associated with irreversible changes in renal structure or physiology.  This assumption is particularly risky in subjects with relatively well preserved renal function and absent or trace proteinuria -- demonstrably the large majority of subjects in the authors' cohort and evidently the primary focus of their broader project.  In these subjects, the likelihood of a transient reduction of eGFR due to any episode leading to dehydration may well be greater than of an event causing an irreversible loss of function (conflicting with the assumption that "true" [hidden] eGFR cannot increase following a drop in eGFR).  Further, subjects with prior episodes of AKI are known to have greater subsequent chance of progressing to ESRD, and subjects receiving treatment with any of the three classes of treatment shown to be effective in delaying progression (renin/angiotensin/aldosterone inhibitors, reduction of systolic blood pressure, and more recently SGLT2 inhibitors) are all associated with immediate reductions of eGFR that are associated with later slower risk of subsequent progression.  These facts conflict with the assumption that past changes in eGFR leading to a Markov stage transition are not informative of the chance of subsequent loss of eGFR.  I suggested using an alternative model -- joint modeling -- that deals similarly with the issue of errors in measurement of proposed predictors by estimating a true but "hidden" path of eGFR over time from the observed values and uses estimates from that model at relevant time points as the estimated "true" current value for the joint survival model.  This technique permits use of all the continuous primary data without collapsing it into categories, makes no assumption excluding increases of eGFR, and permits many options for assumptions about the impact of earlier values. Additionally, this approach would permit direct evaluation of the concordance and the rate of classification error of the relevant primary outcomes -- ESRD or death. In my Version 1 report, I noted that "Given the huge amount of information available for this analysis, the inefficiency of the hidden Markov model may be overcome in the analysis of the overall community estimates of rate of progression."  This seems to be the case in the paper cited in their response to my critique, and I agree that their analytic method is useful for evaluation of choice of overall or group policy.  I suspect, though, that alternative analytic methods permitting use of all of the continuous data might be more powerful in refining predictions, and therefore permitting individualized recommendations or guidelines applicable to specific patients.  

 In their response to my Version 1 report, the authors' have included new text recognizing the limitations of the Markov approach and recognized that "Further research could include assessing the impact of the Markov assumption on predicting kidney function decline using HMMs."  Given this addition, I now approve this manuscript.

Is the work clearly and accurately presented and does it cite the current literature?

Yes

If applicable, is the statistical analysis and its interpretation appropriate?

Partly

Are all the source data underlying the results available to ensure full reproducibility?

No

Is the study design appropriate and is the work technically sound?

Partly

Are the conclusions drawn adequately supported by the results?

Partly

Are sufficient details of methods and analysis provided to allow replication by others?

Partly

Reviewer Expertise:

Nephrology, Epidemiology, Statistics, Clinical trial design, performance, and analysis.

I confirm that I have read this submission and believe that I have an appropriate level of expertise to confirm that it is of an acceptable scientific standard.

# Peer review: Reviewer response for version 2

**DOI:** 10.5256/f1000research.136788.r146904

**Published:** 2022-08-23

**Authors:** Dorothea Nitsch

**Affiliations:** 1 Department of Non-Communicable Disease Epidemiology, London School of Hygiene and Tropical Medicine (LSHTM), London, UK

I have no further comments.

Is the work clearly and accurately presented and does it cite the current literature?

Partly

If applicable, is the statistical analysis and its interpretation appropriate?

Partly

Are all the source data underlying the results available to ensure full reproducibility?

No

Is the study design appropriate and is the work technically sound?

No

Are the conclusions drawn adequately supported by the results?

Partly

Are sufficient details of methods and analysis provided to allow replication by others?

Yes

Reviewer Expertise:

Renal epidemiology

I confirm that I have read this submission and believe that I have an appropriate level of expertise to confirm that it is of an acceptable scientific standard.

# Peer review: Reviewer response for version 1

**DOI:** 10.5256/f1000research.22225.r69718

**Published:** 2020-08-28

**Authors:** Lawrence Hunsicker

**Affiliations:** 1 Department of Internal Medicine, University of Iowa, Iowa City, USA

The authors of this manuscript report on the long-term rate of progression of chronic kidney disease (CKD) in a very large cohort of UK general practice patients followed as part of the Clinical Practice Research Datalink in the time between 1 April 2005 and 31 March 2014. Progression of kidney disease over each patient's course was assessed as the rate of progression from one stage to the next across the six currently standard KDOQI/KDIGO "stages" of renal insufficiency (Stages 1, 2, 3a, 3b, 4, and 5), which are defined by ranges of estimated glomerular filtration rate (eGFR). The rate of progression from one stage to the next was modeled as a Hidden Markov Model, which assumes that subjects are at "true" eGFR stages at each evaluation point, but that these stages may be misclassified based on random error of the eGFR estimate, so that the "observed" transitions may be misidentified. The overall conclusions of the authors are: a) that progression of renal disease to renal failure occurs quite slowly in general, with only a small fraction of patients that progress to kidney failure, and b) that progression of kidney disease as evidenced by [exclusive] use of progression in the KDOQI/KDIGO Stage of CKD, though generally reflective of the average course of kidney disease in the community, is associated with significant error when applied to an individual. Though the authors don't say this, the implication is that use of their analysis may not be very helpful in establishing guidelines as to in whom, and at what repeated intervals, eGFR determinations would be cost effective.

 The major strength of this study is the almost unique test bed offered by access to the data of almost 2,000,000 UK qualified general practice patients from Clinical Practice Research Datalink with "up-to-standard" data collection. Given the choice of analytic method, the authors' analysis is technically entirely competent. The assumptions and methods are well described. The results are clearly explained and put in appropriate context in the Discussion Section. The major limitation of the available data is that patients with the more advanced Stages of kidney disease are likely to have been referred to specialist providers, so that data on the more advanced stages of kidney disease, where progression is generally both more rapid and more clinically important, are more limited and more susceptible to bias. The authors clearly acknowledge this. Further, the issue addressed by this manuscript is potentially important, at least from a health economics point of view. Current policy recommendations uniformly encourage periodic assessment of renal function "in people with, or at risk of, chronic kidney disease (CKD)." But as noted above, in general kidney disease progresses quite slowly and only a small minority of subjects even with documented kidney disease ever progress to chronic renal failure. There is almost no empirical data upon which to make any data-based recommendation about in whom or how often the "periodic assessments" should be done. 

 But the choice of analytic method is subject to criticism. The continuous variable eGFR may range from over 100 (mL/min/1.73 M ^2^) to close to 0. The reduction of this range of values to a classification with 6 levels is associated with massive information loss. Markov models deal with chances of transition from one state to another, and all members of one state are considered to be interchangeable, so that a patient with an eGFR of 100 (at Stage 1/2 with the combination of these two stages) is treated as equivalent to one with an eGFR of 62. But surely the chance of progression from an eGFR of 100 to an eGFR < 60 (Stage 3a) is much lower than the chances of progression from a value of 62 to < 60 . Similarly, it is intuitively likely (though not at all certain) that a subject with a history of rapid loss of eGFR in the past will have more rapid progression in the future. But with a Markov model, all history is lost, and only the current stage, and not the prior history is taken into account. Given the huge amount of information available for this analysis, the inefficiency of the hidden Markov model may be overcome in the analysis of the overall community estimates of rate of progression. But these results will be of almost no use in prediction of the progression of kidney disease in the individual patient. And it is the individual patient's progress, not the average rate of progression in the community, that we are trying to clarify in recommending individual testing eGFR and setting repeat testing intervals. 

 An alternative approach would have been to use a hierarchical ("mixed") analytic method (or perhaps even better, joint analysis combining the hierarchical analysis with a time to event analysis for ESRD and death) to evaluate the overall community rate of progression while estimating the distribution of individual rates of progression. Mixed models deal automatically with the problem that eGFR is measured with error (whether due to variability of the underlying "true" GFR, error due to the misestimation of GFR by eGFR -- up to 35% CKD Stage misclassification using the CKD-Epi formula: Levey *et al.* (2009) ^1^, error due to the underlying determination of serum creatinine (Scr) which is increased at low levels of Scr because of its inverse relationship with eGFR, or variability in timing of the underlying measurements of serum creatinine). Mixed methods also permit specification of alternative models of change over time, permitting exploration of the hypotheses that progression is not linear. They would also facilitate development of time dependent models, allowing input of changes to patient's diabetes, hypertension, and cancer status over time, rather than limiting those variables to the baseline analysis. Most important, this sort of analysis might permit identification of the subset of individuals for whom the risk of progression is the highest -- that might be most benefited by careful follow-up of eGFR, and might give better guidance as to cost-effective testing intervals.

 The authors' choice of analytic methods in this analysis should be put into perspective. The authors were evidently motivated to undertake this analysis so that cost-effectiveness studies could be done specifically in terms of the KDOQI/KDIGO CKD staging mechanism -- presumably to make it easier to develop easily followed guidelines for testing frequency. (See the authors' comment and reference 29 in their final Conclusion.) If the authors felt constrained by these requirements, they have performed a reasonable, if inefficient, analysis. But they and the authors of reference 29 might have done better to request and perform a more efficient analysis using the underlying eGFR data rather than the derived CKD Stages to develop their model, and then abstracted stage specific recommendations from the more efficient model, rather than the other way around. 

 In sum, the analysis as performed using the specified analytic model was competently done, and the overall conclusions are justified - that the rate of progression of renal insufficiency is slow on average, and that dependence on this model would be associated with potentially significant error. But these limited conclusions don't exclude the possibility that more clinically reliable and useful data might be obtained by use of a method that didn't throw away so much of the available data.

Is the work clearly and accurately presented and does it cite the current literature?

Yes

If applicable, is the statistical analysis and its interpretation appropriate?

Partly

Are all the source data underlying the results available to ensure full reproducibility?

No

Is the study design appropriate and is the work technically sound?

Partly

Are the conclusions drawn adequately supported by the results?

Partly

Are sufficient details of methods and analysis provided to allow replication by others?

Partly

Reviewer Expertise:

Nephrology, Epidemiology, Statistics, Clinical trial design, performance, and analysis.

I confirm that I have read this submission and believe that I have an appropriate level of expertise to confirm that it is of an acceptable scientific standard, however I have significant reservations, as outlined above.

## References

[1] : A new equation to estimate glomerular filtration rate. *Ann Intern Med* .2009;150(9) : 10.7326/0003-4819-150-9-200905050-00006 604-12 10.7326/0003-4819-150-9-200905050-00006 19414839PMC2763564

# Response

**Published:** 2022-07-27

**Authors:** Benjamin Feakins

**Affiliations:** Xcenda, UK

**Reviewer:** The authors of this manuscript report on the long-term rate of progression of chronic kidney disease (CKD) in a very large cohort of UK general practice patients followed as part of the Clinical Practice Research Datalink in the time between 1 April 2005 and 31 March 2014. Progression of kidney disease over each patient's course was assessed as the rate of progression from one stage to the next across the six currently standard KDOQI/KDIGO "stages" of renal insufficiency (Stages 1, 2, 3a, 3b, 4, and 5), which are defined by ranges of estimated glomerular filtration rate (eGFR). The rate of progression from one stage to the next was modeled as a Hidden Markov Model, which assumes that subjects are at "true" eGFR stages at each evaluation point, but that these stages may be misclassified based on random error of the eGFR estimate, so that the "observed" transitions may be misidentified. The overall conclusions of the authors are: a) that progression of renal disease to renal failure occurs quite slowly in general, with only a small fraction of patients that progress to kidney failure, and b) that progression of kidney disease as evidenced by [exclusive] use of progression in the KDOQI/KDIGO Stage of CKD, though generally reflective of the average course of kidney disease in the community, is associated with significant error when applied to an individual. Though the authors don't say this, the implication is that use of their analysis may not be very helpful in establishing guidelines as to in whom, and at what repeated intervals, eGFR determinations would be cost effective.

**Authors:** We disagree. We believe our model (unlike others that assume the observed eGFR is equal to true eGFR) permits analysis that correctly considers the misclassification that commonly occurs when monitoring kidney function using estimated GFR. This is important because when a person is misclassified into a higher stage treatment may represent a cost but provide less benefit than expected and in the opposite scenario a misclassification into a lower stage (false negative) would not incur costs from direct treatment but would render the patient at a higher risk for longer. Attempts to model the cost-effectiveness of monitoring without taking this into account are potentially misleading. The work presented in this manuscript formed part of a wider research project, which is intended to assess the cost effectiveness of kidney function monitoring. The health economics study using the outputs from this paper can be found at https://doi.org/10.1371/journal.pmed.1003478 . The Health and Technology Assessment programme this work feeds into can be found at https://www.journalslibrary.nihr.ac.uk/programmes/pgfar/RP-PG-1210-12003 .

**Reviewer:** The major strength of this study is the almost unique test bed offered by access to the data of almost 2,000,000 UK qualified general practice patients from Clinical Practice Research Datalink with "up-to-standard" data collection. Given the choice of analytic method, the authors' analysis is technically entirely competent. The assumptions and methods are well described. The results are clearly explained and put in appropriate context in the Discussion Section. The major limitation of the available data is that patients with the more advanced Stages of kidney disease are likely to have been referred to specialist providers, so that data on the more advanced stages of kidney disease, where progression is generally both more rapid and more clinically important, are more limited and more susceptible to bias. The authors clearly acknowledge this. Further, the issue addressed by this manuscript is potentially important, at least from a health economics point of view. Current policy recommendations uniformly encourage periodic assessment of renal function "in people with, or at risk of, chronic kidney disease (CKD)." But as noted above, in general kidney disease progresses quite slowly and only a small minority of subjects even with documented kidney disease ever progress to chronic renal failure. There is almost no empirical data upon which to make any data-based recommendation about in whom or how often the "periodic assessments" should be done.

**Authors:** We would like that thank the Reviewer for the very positive comments and we agree that patients with more advanced stages of kidney disease might have been referred to specialist care. We would like to emphasise that the main aim of this study was estimate the rate of progression of kidney function decline before they enter end-stage renal disease, requiring specialist care. Therefore, there is a plethora of data, on which to make data-based recommendations regarding the frequency of periodic assessments. Tables S17–20 show that where such recommendations have been made, these have ‘only’ been for patients in eGFR stages G3a and G3b. Patients in these stages are still monitored within primary care, as opposed to specialist care. Moreover, as general practices are financially incentivised by the Quality and Outcomes Framework (QOF) to monitor such patients, there is no shortage of data for these patients as demonstrated in Table 1. Furthermore, it’s also worth noting that Table 1 represents the baseline values for patients from an open cohort spanning the years 2005–2014. During this time, there were two substantial changes to QOF that increased the frequency of serum creatinine and proteinuria testing—one in 2006 and another in 2009. The effect of these changes can be seen in Figure 3. This figure shows that serum creatinine testing increased in subjects with CKD stages ≥2 from 2005 to 2008, where it reached a new plateau, and proteinuria testing increased in subjects with CKD stages ≥4 from 2008 to 2010, where it too reached a new plateau. A significant number of patients would have entered our study cohort prior to these dates, making Table 1 a poor reflection of the general quality of the testing data https://doi.org/10.1136/bmjopen-2018-028062. This figure shows that serum creatinine testing increased in subjects with CKD stages ≥ 2 from 2005 to 2008, where it reached a new plateau, and proteinuria testing increased in subjects with CKD stages ≥ 4 from 2008 to 2010, where it too reached a new plateau. A significant number of patients would have entered our study cohort prior to these dates, making Table 1 a poor reflection of the general quality of the testing data.

**Reviewer:** But the choice of analytic method is subject to criticism. The continuous variable eGFR may range from over 100 (mL/min/1.73 m ^2^) to close to 0. The reduction of this range of values to a classification with 6 levels is associated with massive information loss. Markov models deal with chances of transition from one state to another, and all members of one state are considered to be interchangeable, so that a patient with an eGFR of 100 (at Stage 1/2 with the combination of these two stages) is treated as equivalent to one with an eGFR of 62. But surely the chance of progression from an eGFR of 100 to an eGFR < 60 (Stage 3a) is much lower than the chances of progression from a value of 62 to < 60. Similarly, it is intuitively likely (though not at all certain) that a subject with a history of rapid loss of eGFR in the past will have more rapid progression in the future. But with a Markov model, all history is lost, and only the current stage, and not the prior history is taken into account. Given the huge amount of information available for this analysis, the inefficiency of the hidden Markov model may be overcome in the analysis of the overall community estimates of rate of progression. But these results will be of almost no use in prediction of the progression of kidney disease in the individual patient. And it is the individual patient's progress, not the average rate of progression in the community, that we are trying to clarify in recommending individual testing eGFR and setting repeat testing intervals.

**Authors:** The Reviewer is correct in stating that the Markov assumption means that each subject’s transition to a more severe eGFR stage depends only upon their current stage. We agree that this assumption may be an oversimplification of reality, as it would be likely that an individual who has progressed rapidly through previous stages will continue to do so, and vice versa. As for the reviewers point about likelihood of progression being different if eGFR was 100 vs 62 ml/min/1.73m ^2^, we do not disagree that progression to a next stage would be different, but we would emphasise the problem of knowing what true eGFR is. For example, someone in whom true eGFR is 80 ml/min/1.73m ^2^, their observed eGFR could be anywhere between 70 and 90 ml/min/1.73m ^2^. So, by categorising eGFR into six levels there is some loss in information, but we have a more robust inference. We strongly disagree that these models will be almost of no use. Our models provide average progression times conditional on eGFR stage and presence of albuminuria, and other co-morbidities, e.g., heart failure. The same problem would exist for mixed effect models in as much as you can only provide aggregated rates of decline. We have added the following paragraph to the Discussion section:

 “ *We could be criticised for using an approach that categorises kidney function rather than a method that models continuous eGFR, such as generalised linear mixed models. This is because categorisation can lead to loss of information and reduced statistical power. However, such information loss is typically small when the number of categories is large [Altman D and Royston P, 2006], as was the case in our study. Furthermore, the use of categories that naturally aligned with clinically meaningful eGFR stages added to the interpretability of our findings. In addition, HMMs assume that all individuals within a state are interchangeable, and that the chance of progression to subsequent states depends only on the current state. This assumption may not hold if a patient whose kidney function has previously rapidly declined continues along this trajectory. To mitigate this, we have included updated risk factor information in our model and stratified by baseline albuminuria status. Further research could include assessing the impact of the Markov assumption on predicting kidney function decline using HMMs.”*

**Reviewer:** An alternative approach would have been to use a hierarchical ("mixed") analytic method (or perhaps even better, joint analysis combining the hierarchical analysis with a time to event analysis for ESRD and death) to evaluate the overall community rate of progression while estimating the distribution of individual rates of progression. Mixed models deal automatically with the problem that eGFR is measured with error (whether due to variability of the underlying "true" GFR, error due to the misestimation of GFR by eGFR -- up to 35% CKD Stage misclassification using the CKD-Epi formula: Levey *et al.* (2009) ^1^, error due to the underlying determination of serum creatinine (Scr) which is increased at low levels of Scr because of its inverse relationship with eGFR, or variability in timing of the underlying measurements of serum creatinine). Mixed methods also permit specification of alternative models of change over time, permitting exploration of the hypotheses that progression is not linear. They would also facilitate development of time dependent models, allowing input of changes to patient's diabetes, hypertension, and cancer status over time, rather than limiting those variables to the baseline analysis. Most important, this sort of analysis might permit identification of the subset of individuals for whom the risk of progression is the highest -- that might be most benefited by careful follow-up of eGFR and might give better guidance as to cost-effective testing intervals.

**Authors:** The reviewer is correct, there are alternative statistical models to the approach we led with in this paper. Our initial intention was to model eGFR (or log eGFR) using mixed effect models, but we decided against it for two reasons: the trajectories of eGFR are not linear or necessarily even monotonic in time; more flexible modelling is limited by the fact that for many people only a few eGFR measurements existed in their health record. The models that we did fit did not meet the assumptions of normality implicit in the random effects approach and this, we believe, led to poor predictive performance of these models. It is possible that in an alternative data set, it would be possible to fit these models, but this was not our experience using routinely collected GP data. We would like to stress that we used updated covariates in all the HMMs used in this study and did not condition on only the baseline values of these.

**Reviewer:** The authors' choice of analytic methods in this analysis should be put into perspective. The authors were evidently motivated to undertake this analysis so that cost-effectiveness studies could be done specifically in terms of the KDOQI/KDIGO CKD staging mechanism -- presumably to make it easier to develop easily followed guidelines for testing frequency. (See the authors' comment and reference 29 in their final Conclusion.) If the authors felt constrained by these requirements, they have performed a reasonable, if inefficient, analysis. But they and the authors of reference 29 might have done better to request and perform a more efficient analysis using the underlying eGFR data rather than the derived CKD Stages to develop their model, and then abstracted stage specific recommendations from the more efficient model, rather than the other way around.

**Authors:** The reviewer is not entirely correct. This model was one part of a larger funded project which included a cost-effectiveness study using the outputs from this model, but it is not true that we felt or were constrained by this. Our initial plan was to model eGFR as a continuous measure, but we did not for reasons explained in the previous response. We feel the objective of our work is quite the opposite to the reviewer and we would prioritise models than fit clinical practice over and above statistical efficiency.

**Reviewer:** In sum, the analysis as performed using the specified analytic model was competently done, and the overall conclusions are justified - that the rate of progression of renal insufficiency is slow on average, and that dependence on this model would be associated with potentially significant error. But these limited conclusions don't exclude the possibility that more clinically reliable and useful data might be obtained by use of a method that didn't throw away so much of the available data.

**Authors:** We disagree with the assertion made by the reviewer that our method “threw so much of the data away”. We agree that categorisation can result in loss of information, but this is most likely to occur when continuous measures are split in two (dichotomisation) and applied to small data sets. The thresholds for categorisation should also not be defined post-hoc. Neither of these are true in our case. To cite Altman and Royston “using multiple categories (to create an “ordinal” variable) is generally preferable to dichotomising. With four or five groups the loss of information can be quite small, but there are complexities in analysis” (see https://doi.org/10.1136/bmj.332.7549.1080 ). Moreover, we have provided calibration plots in Figures S1–4 that demonstrate the accuracy of the predictions made by the models.

# Peer review: Reviewer response for version 1

**DOI:** 10.5256/f1000research.22225.r53695

**Published:** 2019-09-11

**Authors:** Dorothea Nitsch

**Affiliations:** 1 Department of Non-Communicable Disease Epidemiology, London School of Hygiene and Tropical Medicine (LSHTM), London, UK

The authors used CPRD records to inform a multistate model on progression of kidney disease. They appropriately considered that there could be measurement error and allowed for this to some extent. 

 They used outcome variables that have not been validated for incident analyses in CPRD(dialysis/transplantation).

 The underlying model for 'true' but unobserved kidney disease assumed that kidney disease could only get worse over time but not better - but this is not supported by actual data which suggest that transient decreases can improve over time and may not necessarily need to be permanent (though such changes may be a risk marker for later decline). Here the authors should discuss more about the chronicity assumption within the CKD definition and how they parametrised this in their data.

 Then there was a model for measurement error, but this seems to be a static model, i.e. assuming that measurement error does not vary over time - did I understand this correctly? Because over time in the UK the way creatinine gets measured and reported has changed dramatically during the study period. This change in reporting of calibrated creatinines in effect hides progression over time when crude analyses are used as different labs shifted to calibration and reporting of creatinine to IDMS at different points in time - thereby shifting the entire creatinine distribution down by 5% over the years prior to 2014 (i.e. hiding a decrease in kidney function over time).

 Did the authors recalculate eGFR from the creatinines (which then requires some thought about time-dependent measurement error which varies by lab and time) - or use reported eGFRs (which then means they lost a lot of measurements based on thresholds with informative missingness as eGFR does not get reported uniformly - some labs only report values in a range of >15 and <60, others report up to 90ml/min/1.73m2)? Dependent on whether the authors used creatinine or eGFR they have discussed further biases in their design and explicitly allow for such biases in their model.

 Then there is the overall study design which is a somewhat odd cohort as it is dependent on having three or more eGFR (or creatinine?) tests and that is not representative of the general population - the survey and CPRD validated prevalence of reduced eGFR in the UK population is about 6% and here the numbers are much higher (3-7 fold depending on albuminuria category) simply because these represent an enriched sample as GPs had a reason to test more than once but indeed three times. Does this enriched sample really represent 'a model for kidney disease progression'? The authors should discuss this and whether people who should have been tested but weren't tested may be a high risk group. The sample here represents a group of patients who engage with the health service but the people who progress in truth may be not fully captured. This needs to be discussed more.

 There were real financial reasons for testing e.g. annual testing for diabetes (started in 2004), and less so for other illnesses, but risk factors for CKD determine testing rates and especially repeat testing as reported by the UK National CKD Audit, and there is some understanding from this audit how testing schemes are carried out. So I would disagree with "A major strength of this study is that we have taken a very large and unselected sample of patients from a database that has been shown to be representative of the wider UK population" as stated in the discussion - this is a selected sample and not representative of what happens overall in terms of kidney function decline as not all are tested the same way. I would have stratified by underlying comorbidity and not simply adjusted for it.

 I totally agree with the authors about the selective loss to follow-up with loss of people who are managed by other specialities including renal in secondary care. 

 Overall this is an interesting analysis, but more work is needed to convince me that this model should be used for economic modelling.

Is the work clearly and accurately presented and does it cite the current literature?

Partly

If applicable, is the statistical analysis and its interpretation appropriate?

Partly

Are all the source data underlying the results available to ensure full reproducibility?

No

Is the study design appropriate and is the work technically sound?

No

Are the conclusions drawn adequately supported by the results?

Partly

Are sufficient details of methods and analysis provided to allow replication by others?

Yes

Reviewer Expertise:

Renal epidemiology

I confirm that I have read this submission and believe that I have an appropriate level of expertise to confirm that it is of an acceptable scientific standard, however I have significant reservations, as outlined above.

# Response

**Published:** 2022-07-27

**Authors:** Benjamin Feakins

**Affiliations:** Xcenda, UK

**Reviewer:** They used outcome variables that have not been validated for incident analyses in CPRD (dialysis/transplantation).

**Authors:** We did not model or attempt to estimate the incidence of dialysis or transplantation. We have included patients who would be candidate for dialysis or transplantation (patients with stage 4/5 CKD), but we censored patients at the first code relating to dialysis or transplantation from hospital or GP records. Moreover, we excluded patients with history of renal replacement therapy. It is unlikely that a significant number of patients would have received either dialysis or renal replacement therapy without there being a code to identify it in the data.

**Reviewer:** The underlying model for 'true' but unobserved kidney disease assumed that kidney disease could only get worse over time but not better - but this is not supported by actual data which suggest that transient decreases can improve over time and may not necessarily need to be permanent (though such changes may be a risk marker for later decline). Here the authors should discuss more about the chronicity assumption within the CKD definition and how they parametrised this in their data.

**Authors**: It is true that kidney function, albeit measured by an imperfect test (e.g., eGFR) has been observed to improve and whilst this might be true, observed improvements in kidney function could also be as a result of measurement error or intervention. For simplicity, and with the agreement of the clinicians on the paper (DL and CO’C), we thought the unidirectional model for true underlying kidney function to be biologically plausible. We did perform a sensitivity analysis in which we allowed underlying kidney function to spontaneously (without intervention) improve. We concluded that this model did not fit the data as well as the progression only model. We have added the following text to the eighth paragraph of the Discussion section:

 “ *The assumption that true underlying kidney function only deteriorates with age is a fundamental part of the model and further research could investigate alternative models for underlying kidney progression and their impact monitoring recommendations.”*

**Reviewer:** Then there was a model for measurement error, but this seems to be a static model, i.e. assuming that measurement error does not vary over time - did I understand this correctly? Because over time in the UK the way creatinine gets measured and reported has changed dramatically during the study period. This change in reporting of calibrated creatinines in effect hides progression over time when crude analyses are used as different labs shifted to calibration and reporting of creatinine to IDMS at different points in time - thereby shifting the entire creatinine distribution down by 5% over the years prior to 2014 (i.e. hiding a decrease in kidney function over time).

**Authors:** This is a valid point, relating to a problem we have encountered in routine health record data sets. However, we feel that this issue is likely to have minimal impact to our study, as apart from the last 3.25 years, our data pre-dates the use of IDMS as the changes took place in 2010. Furthermore, we conducted this study in an open cohort (CRPD-Vision) that has been decreasing in size ( https://doi.org/10.1136/bmjopen-2017-020738 ), further attenuating the pool of potentially affected tests.

**Reviewer:** Did the authors recalculate eGFR from the creatinines (which then requires some thought about time-dependent measurement error which varies by lab and time) - or use reported eGFRs (which then means they lost a lot of measurements based on thresholds with informative missingness as eGFR does not get reported uniformly - some labs only report values in a range of >15 and <60, others report up to 90ml/min/1.73m2)? Dependent on whether the authors used creatinine or eGFR they have discussed further biases in their design and explicitly allow for such biases in their model.

**Authors:** In this study, we used all testing sources available to obtain eGFR, including pre-derived eGFR values. Unfortunately, the data source used provides no information on the techniques used to measure serum creatinine levels or to estimate eGFR. We also no longer have access to this data. Hence, we cannot say whether the calculation of a particular eGFR value was performed using laboratory methods or via an estimating equation in a GP practice. However, we estimated the overwhelming majority of eGFR values from raw serum creatinine values. Hence, any bias present from the use of pre-derived eGFR values is likely to be minimal. The method we used to calculate eGFR from serum creatinine was the CKD-EPI equation. We are aware that better method for the calculation of eGFR exist, however, the one we used would have been the one predominantly in use in UK clinical practice throughout the study period.

**Reviewer:** Then there is the overall study design which is a somewhat odd cohort as it is dependent on having three or more eGFR (or creatinine?) tests and that is not representative of the general population - the survey and CPRD validated prevalence of reduced eGFR in the UK population is about 6% and here the numbers are much higher (3-7 fold depending on albuminuria category) simply because these represent an enriched sample as GPs had a reason to test more than once but indeed three times. Does this enriched sample really represent 'a model for kidney disease progression'? The authors should discuss this and whether people who should have been tested but weren't tested may be a high risk group. The sample here represents a group of patients who engage with the health service but the people who progress in truth may be not fully captured. This needs to be discussed more.

**Authors:** The requirement of three serum creatinine tests was imposed to establish a reliable baseline eGFR status (requiring two tests) and to estimate change over time. We concede that the population present in this sample is not representative of a general UK population so much as a UK general practice population, and we have amended the wording of the fourth paragraph of the Discussion section of the manuscript to reflect this. This reads:

 “ *Inclusion into the study was conditional upon having three or more serum creatinine measurements, but creatinine is commonly measured in UK general and not necessarily for the purpose of monitoring kidney function or diagnosing kidney disease.”*

 … and later in the eighth paragraph of the Discussion section:

 “ *Our study design means that we would also miss patients with ESRD who had not engaged in primary care.”*

 The degree to which this could bias our results is open to some conjecture, and we have also made such comments in the Discussion section of the manuscript. Furthermore, others [Gruger J et al., 1991] who have studied the underlying mechanisms for requesting kidney function testing have concluded that the only mechanism by which testing may bias estimates of transition rates is that of patient-driven testing, which we feel is possible, but unlikely.

**Reviewer:** There were real financial reasons for testing e.g. annual testing for diabetes (started in 2004), and less so for other illnesses, but risk factors for CKD determine testing rates and especially repeat testing as reported by the UK National CKD Audit, and there is some understanding from this audit how testing schemes are carried out. So I would disagree with "A major strength of this study is that we have taken a very large and unselected sample of patients from a database that has been shown to be representative of the wider UK population" as stated in the discussion – this is a selected sample and not representative of what happens overall in terms of kidney function decline as not all are tested the same way. I would have stratified by underlying comorbidity and not simply adjusted for it.

**Authors:** When we wrote the section of text in question, we were referring to the non-selected nature of any one individual appearing in the dataset, i.e., their presence was only determined through virtue of their general practice opting into the database. However, as per our previous response, the population used in this study is still representative of a GP patient cohort, which is the setting in which we anticipate these results to be generalised. We have endeavoured to ensure that our study population was as non-specific as possible, but he nature of our study meant that patients needed to have creatinine measures present in their medical history for us to draw any kind of inference on their kidney function decline.

 We would also add that, contrary to the reviewer’s comments, we have stratified our analysis by the factors most important in determining the rate of kidney function decline, being the initial albuminuria stage. This is in line with the findings of other studies in this area [Chang WX et al., 2016; Inaguma D et al., 2017; Coresh J et al., 2019].
